# Effects of Shellfish and Organic Fertilizer Amendments on Soil Nutrients and Tea Yield and Quality

**DOI:** 10.3390/toxics11030262

**Published:** 2023-03-12

**Authors:** Wenbin Liu, Shiyu Cui, Jiawei Ma, Dongtao Wu, Zhengqian Ye, Dan Liu

**Affiliations:** 1 Key Laboratory of Soil Contamination Bioremediation of Zhejiang Province, Zhejiang A & F University, Hangzhou 311300, China; lwb@stu.zafu.edu.cn (W.L.); yezhq@zafu.edu.cn (Z.Y.); 2 The Nurturing Station for the State Key Laboratory of Subtropical Silviculture, Zhejiang A & F University, Hangzhou 311300, China; 3 College of Landscape and Architecture, Zhejiang A & F University, Hangzhou 311300, China; shiyu0408@stu.zafu.edu.cn; 4 Lishui Agricultural and Rural Bureau, Lishui 323000, China; wudongtao1@163.com

**Keywords:** *Camellia sinensis*, heavy metals, soil fertility, tea quality, soil acidification

## Abstract

Soil acidification in tea plantations leads to an excessive heavy metal content in tea, decreasing its yield and quality. How to apply shellfish and organic fertilizers to improve soil and ensure the safe production of tea is still not clear. A two-year field experiment was conducted in tea plantations in which the soil was characterized by a pH of 4.16 and concentrations of lead (Pb) (85.28 mg/kg) and cadmium (Cd) (0.43 mg/kg) exceeding the standard. We used shellfish amendments (750, 1500, 2250 kg/ha) and organic fertilizers (3750, 7500 kg/ha) to amend the soils. The experimental results showed that compared with the treatment without any amendment (CK), the soil pH increased by 0.46 on average; the soil available nitrogen, phosphorus, and potassium contents increased by 21.68%, 19.01%, and 17.51% respectively; and the soil available Pb, Cd, Cr, and As contents decreased by 24.64%, 24.36%, 20.83%, and 26.39%, respectively. In comparison to CK, the average yield of tea also increased by 90.94 kg/ha; tea polyphenols, free amino acids, caffeine, and water extract increased by 9.17%, 15.71%, 7.54%, and 5.27%, respectively; and the contents of Pb, Cd, As, and Cr in the tea decreased significantly (*p* < 0.05) by 29.44–61.38%, 21.43–61.38%, 10.43–25.22%, and 10.00–33.33%, respectively. The greatest effects on all parameters occurred with the largest amendment of both shellfish (2250 kg/ha) and organic fertilizer (7500 kg/ha) combined. This finding suggests that the optimized amendment of shellfish could be used as a technical measure to improve the health quality of both soil and tea in acidified tea plantations in the future.

## 1. Introduction

Tea (*Camellia sinensis* L.) is an important economic crop in China: in 2022, the total area of plantations was 3.2 million ha, yielding 3.2 million t and valued at more than RMB 300 billion [1]. Therefore, we need to continuously study how to improve the yield and quality of tea and the sustainability of the crop. However, the problem of soil acidification is becoming an important factor restricting the development of the tea industry. The main cause of acidification is the excessive application of nitrogen (N) fertilizer. Basker et al. [2] found that the soil acidification caused by N fertilizer application was 25 times that of acid deposition. Yan Peng et al. [3] also showed that the average pH of tea plantation soil in China was 4.73, and more than 52% of plantations had a soil pH below 4.5, which restricted the growth of tea plants (REFS). The acidification of tea plantation soil also increases the uptake of heavy metals such as Pb, Cd, and Chromium (Cr) [4], which may deleteriously affect the health of tea drinkers [5]. In addition, if the soil pH is <4.0, the water-soluble fluorine content can increase rapidly, increasing the uptake of fluorine by tea plants [6].

Soil amendments are one of the important measures to ameliorate acidified soil, as they can effectively improve soil acidity and fertility to recovery the productivity of acidic soil [7]. At present, the common amendments are mainly inorganic, including lime; various shell powders; and industrial by-products (dolomite, fly ash, phosphate rock powder, alkali residue, etc.) [8]. Dimirkou et al. [9] showed that goethite and clinoptilolite had a good adsorption effect on Cd and As. Mohan et al. [10] used biochar formed by the pyrolysis of different types of fast wood/bark to adsorb As, Cd, and Pb. It was found that the adsorption of Pb and Cd by rubber bark biochar was better than that by oak, pine, and pine bark biochar. However, in practical applications, biochar and certain mineral amendments are not widely used because of their high price or the presence of a small amount of heavy metals. Although lime is cheap, the long-term large-scale application of lime hardens the soil, resulting in an imbalance of calcium, magnesium, potassium, and other elements, reducing crop yield [11]. Shellfish such as oysters, scallops, mussels, clams, and conches are the main raw materials in shell amendments for soil. Shell is a natural biomineral material containing a large amount of CaCO_3_. When it is calcined at a high temperature, it generates CaO, which has a strong adsorption and exchange capacity [12]. Studies have shown that shell powder can significantly increase the soil pH and reduce the availability of Cd and the acid soluble content of Cd in soil, increasing the residual content. At the same time, it can effectively reduce the absorption of Cd by plants [13]. It thus reduces, to a certain extent, the bioavailability of heavy metals to crops and the risk to consumers. However, the long-term application of alkaline substances such as soil amendments while neglecting the input of organic fertilizers causes a soil cation imbalance [14]; reduces the availability of trace elements such as Fe and Mn and non-metallic elements such as B; and ultimately affects plant growth [15]. Organic fertilizers can be condensed into new humus by microbial action after being applied to the soil and have a strong binding ability with soil particles [16]. At the same time, organic fertilizers contain trace nutrients, sugars, amides, and amino acids, which can promote the growth of plant roots and improve crop quality [17]. Ji et al. [18] found that after the application of organic fertilizers in a tea plantation, the tea polyphenol, free amino acid, and caffeine contents increased significantly. Therefore, the rational use of soil amendments and organic fertilizers is an important measure to improve tea plantation soil and tea quality.

Despite the foregoing research, it remains unclear how to maintain the nutrient balance and reduce tea safety risks in the process of improving soil quality. In this study, an acidified tea plantation with excessive Pb and Cd was selected to carry out a two-year field experiment to examine the effects of shellfish amendments and organic fertilizers on soil nutrients, tea heavy metals, and tea yield and quality. We hoped to provide a scientific basis for the future use of shellfish and organic fertilizer amendments in soils to improve acidified tea plantation soil, ensuring the safe production of tea.

In this research, we addressed the following scientific hypotheses: (i) the application of a shellfish amendment and organic fertilizer could improve tea plantation soil nutrients; (ii) a high dosage of shellfish amendment and organic fertilizer could significantly reduce the content of available heavy metals in tea and soil; and (iii) the combination of a shellfish amendment and organic fertilizer could improve the yield and quality of tea.

## 2. Materials and Methods

### 2.1. Experimental Area and Soil Properties

The test site was located in a tea plantation in Lishui City, Zhejiang Province, China. This area exceeded China’s “Soil Environmental Quality Agricultural Land Soil Pollution Risk Control Standard “ (GB 15618-2018) (when soil pH ≤ 5.5, Cd < 0.3 mg/kg, and Pb < 70 mg/kg). At the same time, it was found that the Pb content in some tea samples in this area exceeded the national food safety standard of China (GB 2762-2017) (Pb < 5 mg/kg). The area has a subtropical monsoon climate with four distinct seasons; it is warm and humid and experiences abundant rainfall, a long winter and summer, a short spring and autumn, and high temperatures. Due to the complex terrain, there is a vertical climate with an altitude disparity. The annual average temperature is about 18.3 °C; the extreme minimum temperature is 1.8 °C; the extreme maximum temperature is 37 °C; and the annual precipitation is 1824.8 mm. The annual average sunshine period is 1510.2 h. The tea variety was ‘Longjing 43’ with a plant age of 8 y. The tea plants were planted in double rows with a row spacing of 1.6 m, and the crop was picked manually. Before the start of the experiment, we used a wooden shovel to collect 0–20 cm soil in the study area, because this depth is usually considered to be the tillage layer, and it is also the key depth for plant roots to absorb nutrients. By collecting and testing bottom soil, we could better evaluate the experimental results and ensure the scientificity of this research. The soil type was Inceptisol, a kind of soil with poor fertility inherited from the parent material and obvious soil structure and color changes. The physical and chemical properties of the soil are shown in Table 1.

### 2.2. Experimental Materials

The shellfish amendment used in the experiment came from Fujian Mata Ecological Technology Co., Ltd. (Xiamen, China). The main raw material was oyster shell. The basic properties were a pH of 9.26, a particle size of 1.00–4.75 mm, a CaO content of 41.15%, a MgO content of 6%, a Na content of 0.88%, a S content of 0.47%, and a Cl content of 0.93%. The organic fertilizer was from Jinhua Huijun Agricultural Co., Ltd. (Jinhua, China). The main raw material was cow dung. The basic properties were a pH of 6.92, an organic matter content of 30.19%, N + P_2_O_5_ + K_2_O = 6%, a total N content of 1.33%, a total P content of 3.32%, and a total K content of 1.34%. The content of heavy metals in the experiment materials in Table 2.

### 2.3. Experimental Design

There were seven treatments, named according to the amount of shellfish amendment and organic fertilizer applied, as shown in Table 3. The shellfish amendment and organic fertilizer were applied in November 2020 and repeated in November 2021. In order to ensure the scientific nature of the test, the plots without any fertilizer and soil amendments were selected for the test within 2 years to avoid the interference of other factors on the experiment. A randomized block design was used in the experiment. Each treatment was repeated three times. The plot area was 80 m^2^ (40 m in length and 2 m in width), and protective rows with an interval of 1.5 m were set in different cells. The chemical fertilizers used for the tea were a compound fertilizer (N:P_2_O_5_:K_2_O = 15:15:15) and urea. The base fertilizer was applied with the compound fertilizer (750 kg/ha) around November every year, and the top dressing was applied three times in February (compound fertilizer at 450 kg/ha + urea at 300 kg/ha), April (compound fertilizer at 450 kg/ha + urea at 150 kg/ha) and July (compound fertilizer at 450 kg/ha). The shellfish amendment and fertilizer were applied by strip. Along the tea plant row in the plot, 2/3rds of the way from the root zone of the tea plant to the edge of the crown (about 0.3 m from the tea plant), a 20 cm wide and 15 cm deep fertilization ditch was opened and covered with soil after fertilizer application. Other field management measures (weeding, watering, spraying pesticides, pruning, etc.) were consistent with the local practices.

### 2.4. Sampling

Tea samples: in March 2022, sampling was conducted during the spring tea picking season. The picking standard was one bud and two leaves of fresh tea. Immediately after picking, the harvested material was taken back to the laboratory and placed in a preheated oven at 105 °C for 10–15 min, then dried to a constant weight at 80 °C. The dry samples were stored in a refrigerator (4 °C) until tested.

Soil samples: in March 2022, the surface soil (0–20 cm) was collected using a wooden shovel in the root zone of the tea plants using a 5-point sampling method. After the removal of the impurities (gravel, tea leaves, and non-soil components), the soil was fully mixed and separated by the quartering method. Each sample weighed 1 kg and was naturally air-dried and then ground and passed through 2 mm and 0.149 mm sieves to determine soil physical and chemical properties and soil heavy metals.

### 2.5. Determination of Soil Physical and Chemical Properties

Soil pH was determined using 10 mM CaCl_2_ for extraction and a pH meter (Orion 3 Star, Thermo Ltd., Waltham MA, USA) (soil:liquid = 1 g:2.5 mL) [19]. Soil available P was determined using HCl-NH_4_F for extraction and the molybdenum blue method [20]. Available N was determined by the alkaline hydrolysis diffusion method [21]; available K was determined by CH_3_COONH_4_ extraction and flame photometry [22]; and organic carbon was determined by the K_2_Cr_2_O_7_ oxidation capacity method and external heating method [23] (soil organic matter = soil organic carbon ∗ 1.724). The total Pb and Cd in the soil were extracted by the HNO_3_-HF-HClO_4_ method, and available Cd, Pb, and Cr in the soil were extracted with 0.1 M HCl (soil/liquid = 1 g:5 mL, extracted for 2 h) and determined by atomic absorption spectrophotometry (AA-7000, Shimadzu, Kyoto, Japan) [24]. The available As was determined by atomic fluorescence spectrophotometry using 0.05 M NH_4_H_2_PO_4_ extraction (soil/solution = 1 g:25 mL, extraction 16 h) (AFS-230E, Haiguang, Beijing, China) [25]. The analytical quality control was carried out with Chinese national standard substance GBW07442 (GSF-2), and the blank samples and parallel samples were determined. The results showed that the contents of Cd, Pb, Cr, and As met the allowable error values. 

### 2.6. Determination of Tea Yield, Quality, and Heavy Metals

The determination of the 100-bud weight (fresh weight of one bud and two leaves of sufficient new shoots) in each plot: 100 new shoots were randomly selected and weighed, and this was repeated 6 times. Tea yield was measured by the whole plot yield (excluding the protection line), and the yield per ha (kg/ha) was obtained by conversion. Budding density was determined using a 0.1 m^2^ sample box and the 5-point sampling survey method (33 cm × 33 cm): one bud and two leaves in the box were picked from top to bottom, and the budding density was recorded [26]. The measurements for each plot were repeated 6 times. Tea polyphenols were determined by ferrous tartrate colorimetry; total free amino acids were determined by ninhydrin colorimetry; water extract content was determined by the water bath extraction drying method; caffeine content was determined by ultraviolet spectrophotometry; and the phenol ammonia ratio was recorded as tea polyphenols/total free amino acids [27]. The contents of N, P, and K in the tea were digested by the H_2_SO_4_-H_2_O_2_ combined digestion method. The N content in the samples was determined by a Kjeldahl apparatus (NKY6120, Shanghai, China) [28]. The P content in the samples was determined by vanadium molybdenum colorimetry [29]. The K content in the samples was determined by a flame photometer (FP640, Shanghai, China) [30]. A Chlorophyll Meter Model SPAD-502 (Konica Minolta Inc. Made in Tokyo, Japan) was used to determine the SPAD value of the same part of the tea (i.e., the third tea leaf down from the new bud) [31]. The tea samples were digested by the HNO_3_-H_2_O_2_ microwave digestion method (ETHOS UP, Milestone, Sorisole, Italy), and the contents of Pb, Cr, Cd, and As were determined by inductively coupled plasma mass spectrometry (iCAP-Q ICP-MS, Thermo Scientific, Waltham, MA, USA) [32].

### 2.7. Statistical Analysis

Non-parametric statistics and one-way analysis of variance (one-way ANOVA) were performed using IBM SPSS Statistics 23 software. The Duncan method was used to make multiple comparisons within the experimental data. The significance level for the differences was *p* < 0.05. The picture was completed by Origin 2021. The experimental data are expressed as mean ± standard deviation (SD).

## 3. Results

### 3.1. Soil Physical and Chemical Properties

As shown in Table 4, the soil pH of each treatment was 4.08–4.68. Compared with the CK treatment, the pH of the TF2, TF4, and TF6 treatments significantly increased by 0.54, 0.47, and 0.60 units, respectively (*p* < 0.05). Compared with the CK treatment, the soil available P content of the TF3, TF4, and TF6 treatments was significantly increased by 21.54 mg/kg, 22.66 mg/kg, and 24.78 mg/kg, respectively, corresponding to increases of 25.70%, 27.04%, and 29.57% (*p* < 0.05). The soil available K content of the TF6 treatment was the highest, at 160.33 mg/kg. Compared with the CK treatment, the TF4, TF5, and TF6 treatments significantly increased the soil available K content by 19.16%, 32.11%, and 35.49%, respectively (*p* < 0.05). Compared with the CK treatment, the organic matter content of the TF2 and TF6 treatments increased by 17.58% and 19.19%, respectively (*p* < 0.05). The content of available N in the soil was the highest in the TF6 treatment (234.08 mg/kg), being significantly increased by 36.48% (*p* < 0.05) compared with the CK treatment.

### 3.2. Soil Heavy Metals

**Figure 1:**
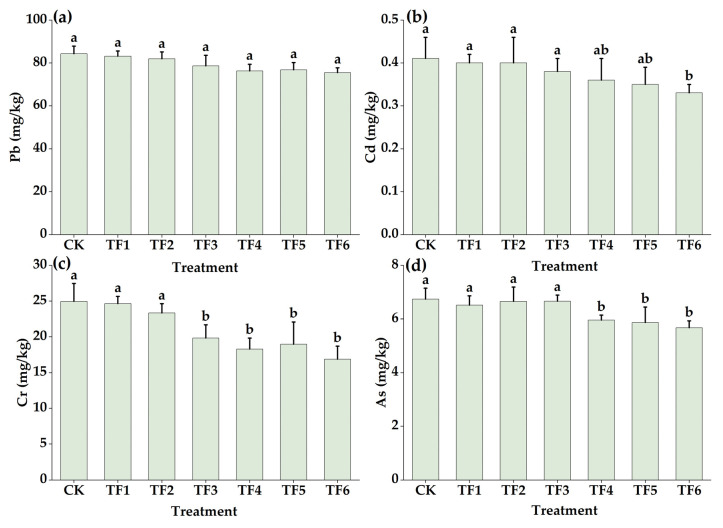
Effects of shellfish amendment and organic fertilizer on soil Pb (**a**), Cd (**b**), Cr (**c**), and As (**d**). Note: no shellfish amendment or organic fertilizer (CK), shellfish amendment 750 kg/ha + organic fertilizer 3750 kg/ha (TF1), shellfish amendment 750 kg/ha + organic fertilizer 7500 kg/ha (TF2), shellfish amendment 1500 kg/ha + organic fertilizer 3750 kg/ha (TF3), shellfish amendment 1500 kg/ha + organic fertilizer 7500 kg/ha (TF4), shellfish amendment 2250 kg/ha + organic fertilizer 3750 kg/ha (TF5), shellfish amendment 2250 kg/ha + organic fertilizer 7500 kg/ha (TF6). Error bars are means ± standard deviation. Different lowercase letters in the figure indicate significant differences between different treatments (*p* < 0.05).

As displayed in Figure 1a, compared with the CK treatment, the soil Pb content of the shellfish amendment and organic fertilizer treatment was not significantly different but showed a trend of a gradual decrease with the increase in dosage. The Pb content of the TF6 treatment was the lowest at 75.48 mg/kg, which was 8.76 mg/kg lower than that of the CK treatment, representing a decrease of 10.40; thus, the difference was not significant (*p* > 0.05). As shown in Figure 1b, the Cd content of the TF6 treatment was the lowest at 0.33 mg/kg, which was significantly smaller (by 0.08 mg/kg) than that of the CK treatment, representing a decrease of 19.51% (*p* < 0.05). Compared with the CK treatment, the soil Cr content of the TF3, TF4, TF5, and TF6 treatments decreased by 20.48%, 26.73%, 24.05%, and 32.42%, respectively (*p* < 0.05). As shown in Figure 1d, compared with the CK treatment, the As content in the TF4, TF5, and TF6 treatments decreased by 11.57%, 12.91%, and 15.88%, respectively (*p* < 0.05).

### 3.3. Soil Available Heavy Metals

**Figure 2:**
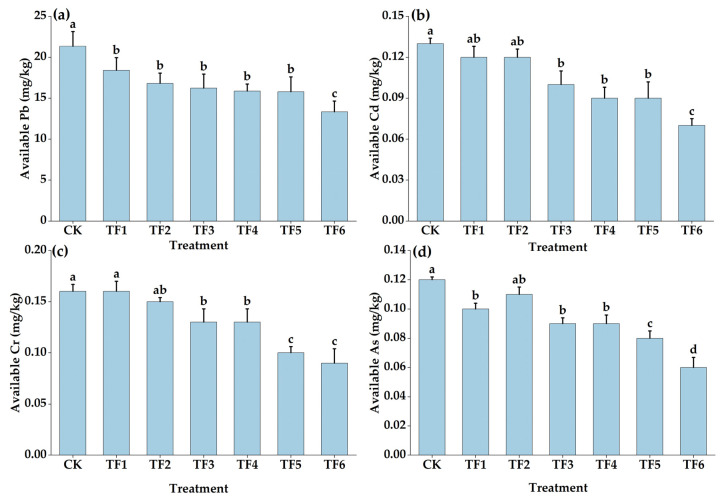
Effects of shellfish amendments and organic fertilizer on soil available Pb (**a**), Cd (**b**), Cr (**c**), and As (**d**). Note: no shellfish amendment or organic fertilizer (CK), shellfish amendment 750 kg/ha + organic fertilizer 3750 kg/ha (TF1), shellfish amendment 750 kg/ha + organic fertilizer 7500 kg/ha (TF2), shellfish amendment 1500 kg/ha + organic fertilizer 3750 kg/ha (TF3), shellfish amendment 1500 kg/ha + organic fertilizer 7500 kg/ha (TF4), shellfish amendment 2250 kg/ha + organic fertilizer 3750 kg/ha (TF5), shellfish amendment 2250 kg/ha + organic fertilizer 7500 kg/ha (TF6). Error bars are means ± standard deviation. Different lowercase letters in the figure indicate significant differences between different treatments (*p* < 0.05).

As shown in Figure 2a, compared with the CK treatment, each treatment significantly reduced the soil available Pb content. At the same time, with the increase in shellfish amendment and organic fertilizer application, the soil available Pb content gradually decreased. The TF6 treatment had the lowest content of 13.35 mg/kg, which was decreased by 37.44% compared with the CK treatment (*p* < 0.05). Compared with the available Pb content before the experiment (20.95 mg/kg), the TF6 treatment decreased by 36.28%. After the application of the shellfish amendment and organic fertilizer, compared with the CK treatment, the soil available Cd content decreased by an average of 24.36%, with the TF3, TF4, TF5, and TF6 treatments presenting significant decreases of 23.08%, 30.77%, 30.65%, and 46.15%, respectively (*p* < 0.05). The content of available Cd in the soil decreased by 28.57% after the experiment. The content of available Cr in the soil was 0.09 mg/kg in the TF6 treatment, which was significantly lower than that in the CK treatment by 43.75%. Compared with the CK treatment, the soil available As content of the TF1, TF3, TF4, TF5, and TF6 treatments decreased by 16.67%, 25.00%, 25.64%, 33.33%, and 50.00%, respectively (*p* < 0.05).

### 3.4. Tea Yield

As shown in Table 5, compared with the CK treatment, the 100-bud weight of the TF2 (25.36 g), TF4 (27.40 g), and TF6 (26.73 g) treatments increased significantly by 14.75%, 23.98%, and 20.95%, respectively. The water content of the tea shoots in each treatment was 0.51–0.54. The budding density of each treatment was 211.33/m^2^–248.33/m^2^ after the application of shellfish amendment and organic fertilizer. Among the treatments, TF6 had the largest budding density, which was significantly increased by 19.08% compared with the CK treatment (*p* < 0.05). The yield of fresh tea was ordered as follows: TF6 (584.02 kg/ha) > TF5 (578.52 kg/ha) > TF2 (561.86 kg/ha) > TF4 (554.45 kg/ha) > TF3 (545.62 kg/ha) > TF1 (483.66 kg/ha) > CK (460.42 kg/ha). Compared with the CK treatment, the shellfish and organic fertilizer treatments increased the yield by 19.75% on average, and the TF5 and TF6 treatments significantly increased the yield by 25.65% and 26.85%, respectively (*p* < 0.05).

### 3.5. Growth and Characteristics of Tea Leaves

As shown in Table 6, the application of the shellfish amendment and organic fertilizer increased the chlorophyll content of the tea, with the highest content resulting from the TF6 treatment (58.29), followed by the TF2 treatment, and then the CK treatment. The N concentration of the tea showed that, compared with the CK treatment, the absorption of N by the tea plants increased by 0.86–7.79% after the application of the shellfish amendment and organic fertilizer, and the highest N concentration was 36.27 g/kg in the TF6 treatment. The P concentration of the tea in each treatment was 2.47–2.86 g/kg, and the P content of the TF6 treatment was the highest, with an increase of 0.39 g/kg compared to the CK treatment, followed by that of the TF1 treatment (2.72 g/kg), which increased by 0.25 g/kg (10.12%). Compared with the CK treatment, the K concentration of the TF1, TF5, and TF6 treatments increased by 5.87%, 8.84%, and 9.66%, respectively (*p* < 0.05).

### 3.6. Tea Quality

As shown in Table 7, the content of polyphenols in the tea was 166.32–197.74 mg/g, and the content of free amino acids was 32.64–41.67 mg/g after the application of the shellfish amendment and organic fertilizer. The tea polyphenols content in the TF5 and TF6 treatments increased significantly by 17.23% and 19.60%, respectively (*p* < 0.05), and the content of free amino acids increased significantly by 8.41 mg/g and 9.49 mg/g, respectively (*p* < 0.05). The results showed that the TF4 treatment had the lowest phenol ammonia ratio (4.72), followed by TF6 (4.74) and TF5 (4.77). Compared with the CK treatment, these ratios were significantly reduced by 0.41, 0.39, and 0.36, respectively. The highest caffeine content was found in the TF6 treatment at 36.53 mg/g, which was significantly increased by 6.12 mg/g compared with the CK treatment, i.e., an increase of 20.12% (*p* < 0.05). The water extract content of each treatment was 398.76–436.28 mg/g, increasing by 2.13–9.41% after the application of the shellfish amendment and organic fertilizer. The water extract content of the TF5 and TF6 treatments significantly increased by 31.85 mg/g and 37.52 mg/g, respectively.

### 3.7. Tea Heavy Metals

**Figure 3:**
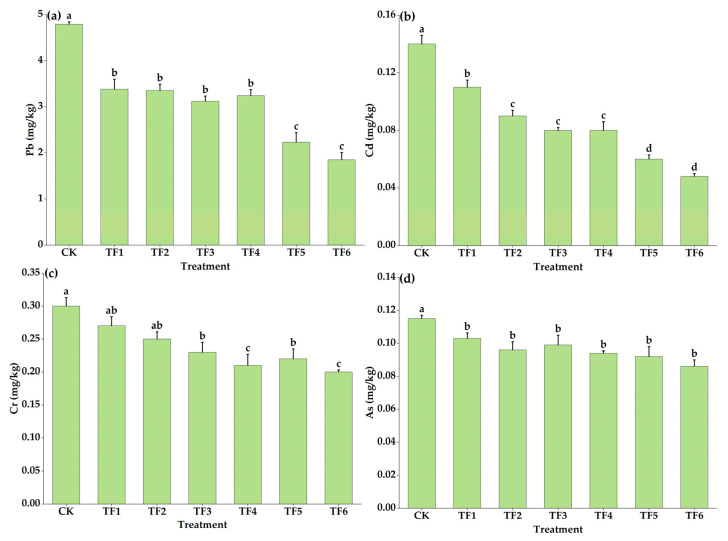
Effects of shellfish amendment and organic fertilizer on Pb (**a**), Cd (**b**), Cr (**c**), and As (**d**) in tea. Note: no shellfish amendment or organic fertilizer (CK), shellfish amendment 750 kg/ha + organic fertilizer 3750 kg/ha (TF1), shellfish amendment 750 kg/ha + organic fertilizer 7500 kg/ha (TF2), shellfish amendment 1500 kg/ha + organic fertilizer 3750 kg/ha (TF3), shellfish amendment 1500 kg/ha + organic fertilizer 7500 kg/ha (TF4), shellfish amendment 2250 kg/ha + organic fertilizer 3750 kg/ha (TF5), shellfish amendment 2250 kg/ha + organic fertilizer 7500 kg/ha (TF6). Error bars are means ± standard deviation. Different lowercase letters in the figure indicate significant differences between different treatments (*p* < 0.05).

As shown in Figure 3a, the Pb content in the tea decreased significantly after the application of the shellfish amendment and organic fertilizer, with a decrease of 29.44–61.38%. When the amount of soil amendment was 2250 kg/ha, the Pb content in the tea decreased most significantly. Compared with the CK treatment, the TF5 and TF6 treatments significantly decreased the Pb content by 2.56 mg/kg and 2.94 mg/kg, respectively (*p* < 0.05). As shown in Figure 3b, with the increase in shellfish amendment and organic fertilizer, the Cd content in the tea gradually decreased, ranging from to 0.11 mg/kg to 0.05 mg/kg. Compared with the CK treatment, the TF6 treatment had the largest decrease of 65.71%, followed by the TF5 treatment with 57.14%. The content of Cr in the tea for TF3, TF4, TF5, and TF6 decreased significantly compared to the CK treatment, with reductions of 23.33%, 30.00%, 26.67%, and 33.33%, respectively. The As content in the tea did not change, though compared with the CK treatment, the application of the shellfish amendment and organic fertilizer treatment significantly decreased its content by 10.43–25.22% (*p* < 0.05).

## 4. Discussion

The selection of soil pH for tea plants is important, and the most suitable pH range for their growth is 4.5–5.5 [33]. Before the experiment, the soil pH was 4.16, which was substantially lower than the normal growth pH of tea plants. However, when the shellfish amendment and organic fertilizer were applied, the soil pH increased to 4.40–4.70. In this experiment, oyster shell was used as the raw material for the shellfish improver. After high-temperature calcination, the main component was the alkaline substance CaO, which effectively increased the soil pH [34]. Our results also showed that the mixed application of the shellfish amendment and organic fertilizer could significantly increase the soil available P, available K, available N, and organic matter contents, enhancing soil fertility. Previous studies have shown that an increase in soil pH can reduce the fixation of P in soil and promote the activity of P-solubilizing microorganisms to activate the fixed P in the soil, thereby increasing the available P content [35]. The increase in soil available K content was mainly due to the fact that after the application of the shellfish amendment and organic fertilizer, the entry of CaO could promote the mineralization of organic matter and free soil slow-release K [36]. At the same time, the affinity of soil adsorption sites for Ca^2+^ is stronger than for K^+^, which increases the K^+^ content in the soil solution [37]. The results of this study showed that the application of the shellfish amendment could significantly increase the soil available N content, which was consistent with the results of Yang et al. [38]. The main reason may have been that the shell powder improver could increase the soil pH, enhance the activity of soil ammonia-oxidizing bacteria, accelerate the mineralization of soil N [39], improve the decomposition efficiency of organic fertilizer, and increase the SOC content [40].

The growth status of tea plants is a key factor affecting the quality and yield of tea. The results of this experiment showed that the application of the shellfish amendment and organic fertilizer could increase the chlorophyll content in tea; promote the absorption and concentration of N, P, and K nutrients in tea plants; and significantly increase the budding density and yield of tea. The content of N, P, and K is of great significance to the formation of tea quality. Phosphorus plays an important role in improving the aroma and taste of tea [41]. The contents of amino acids and caffeine in tea are also significantly positively correlated with the contents of N and K in plants [42]. When the dosage of shellfish amendment and organic fertilizer was 2250 kg/ha and 7500 kg/ha, respectively, the nutrient status of the tea plants was most favorable. Studies have shown that the application of organic fertilizer in tea plantations can significantly improve the yield and quality of tea compared with conventional chemical fertilizers [43]. Because organic matter is the material basis of soil microorganisms and various nutrients in tea plants, it can not only enhance the ability of soil to retain water and fertilizer, but also have a strong buffering capacity for acids and alkalis, promoting the absorption of mineral nutrients by roots [44] The application of amendments increased the soil pH and reduced the toxicity of H^+^ and Al^3+^ to roots, thereby promoting the growth of tea plants and the budding density and yield of tea [45]. Previous studies have confirmed that the content of amino acids and caffeine in tea is significantly positively correlated with the N content in the soil [46]. An increase in the available N content can balance the relationship between lipid metabolism and aroma substance synthesis and improve the quality of tea [47]. Tea polyphenols and water extract increased with the increase in available P in the soil. In addition, the available K in the soil also promoted the amino acid content of tea [48].

The acidification of tea plantation soil reduces the adsorption capacity of soil for heavy metal ions, leading to the dissolution and release of carbonate- and hydroxide-bound heavy metals, weakening the adsorption and fixation of heavy metals in soil to a certain extent and increasing the activity of some heavy metal elements [49]. This provides conditions for the enrichment of heavy metal elements in tea and increases the possibility of heavy metals in tea exceeding the standard. The results of this study showed that the contents of Pb, Cd, Cr, and As in tea leaves decreased significantly after the application of the shellfish amendment and organic fertilizer. At the same time, the content of heavy metals in leaves decreased gradually with an increase in dosage. The application of the shellfish amendment could increase the soil pH, the negative charge on the soil surface, and the adsorption of heavy metals [50]. Bi et al. [51] found that after the application of oyster shell powder in acidic soil, the soil pH increased, the bioavailability of soil Cd decreased significantly, the passivation effect was obvious, and the calcium needed for acidic calcium-deficient farmland could be supplemented. At the same time, organic fertilizer can change the existing forms of heavy metals in the soil, adsorb and complex with them, and increase the soil cation exchange capacity to enhance their adsorption [52]. Yi et al. [53] found that the available content of heavy metals in the soil decreased significantly after the application of organic fertilizer in a tea plantation. Our results confirmed that the soil available heavy metal content decreased significantly after the experiment, which was consistent with previous studies. With the decrease in soil available Pb, Cd, Cr, and As content, the heavy metals absorbed by the tea plants from the soil decreased, and the content of heavy metals in the tea decreased significantly. Therefore, in the acidified tea plantation soil, the application of shellfish amendment and organic fertilizer is of great significance for the safe production of tea, ensuring the safety of tea drinking and reducing its health risks.

## 5. Conclusions

The results showed that the soil pH and nutrients were significantly increased after the application of the shellfish amendment and organic fertilizer in the acidified tea plantation with excessive soil Pb and Cd content. The contents of available heavy metals in the soil and Pb, Cd, Cr, and As in the tea decreased significantly. At the same time, the treatment increased tea yield, improved tea quality, and increased the tea’s economic value. We found that when the dosage of shellfish amendment was 2250 kg/ha and the dosage of organic fertilizer was 7500 kg/ha, the shellfish amendment had the best effect on improving the acidified tea plantation soil and ensuring the safe production of tea. Nevertheless, there were still some shortcomings to our research results. In the next step, we will study the promotion effect of the application of shellfish and organic fertilizers on the growth of tea plants and the mechanism of the improvement in tea quality, as well as the application effect of some new soil amendments (such as biochar and nanomaterials) in acidified tea plantations.

## Figures and Tables

**Table 1:** Soil physical and chemical properties before experiment.

Index	pH	Available N	Available P	Available K	Organic Matter	Available Pb	Available Cd	Available Cr	Available As	Total Cd	Total Pb
Content	4.16	50.46 mg/kg	75.26 mg/kg	110.64 mg/kg	30.14 g/kg	20.95 mg/kg	0.14 mg/kg	0.15 mg/kg	0.12 mg/kg	0.43 mg/kg	85.28 mg/kg

**Table 2:** Heavy metal content of experimental materials in mg/kg.

Experimental Material	Pb	Cd	Cr	As	Hg
Shellfish amendment	10.80	0.37	3.33	1.72	0.06
Organic fertilizer	13.64	0.26	10.26	4.24	0.26

**Table 3:** The dosage of shellfish amendment and organic fertilizer in each treatment.

Treatment	Dosage of Shellfish Amendment kg/ha	Dosage of Organic Fertilizer kg/ha
CK	0	0
TF1	750	3750
TF2	750	7500
TF3	1500	3750
TF4	1500	7500
TF5	2250	3750
TF6	2250	7500

**Table 4:** Effects of shellfish amendment and organic fertilizer on soil physical and chemical properties (pH; available N, P, and K; and OM).

Treatment	pH	Available Phosphorus (mg/kg)	Available Potassium (mg/kg)	Organic Matter (g/kg)	Available Nitrogen (mg/kg)
CK	4.08 ± 0.24 b	83.81 ± 5.42 b	118.33 ± 16.43 b	37.15 ± 3.21 b	171.50 ± 1.71 c
TF1	4.36 ± 0.31 b	90.21 ± 7.40 ab	130.66 ± 11.02 b	40.13 ± 2.46 b	189.45 ± 2.37 c
TF2	4.62 ± 0.26 a	96.66 ± 22.91 ab	125.66 ± 11.91 b	43.68 ± 1.85 a	212.63 ± 17.06 ab
TF3	4.48 ± 0.33 b	105.35 ± 19.05 a	120.33 ± 14.75 b	39.18 ± 2.08 b	204.42 ± 27.91 b
TF4	4.55 ± 0.25 a	106.47 ± 25.83 a	141.00 ± 10.54 a	42.51 ± 3.15 ab	209.39 ± 6.12 b
TF5	4.53 ± 0.31 b	91.19 ± 9.84 ab	156.33 ± 13.23 a	38.23 ± 1.72 b	202.16 ± 28.83 b
TF6	4.68 ± 0.38 a	108.59 ± 19.93 a	160.33 ± 12.74 a	44.28 ± 2.12 a	234.07 ± 10.05 a

Data represent means ± standard deviation. Different lowercase letters in the table indicate significant differences between different amendment treatments (*p* < 0.05). Note: no shellfish amendment or organic fertilizer (CK), shellfish amendment 750 kg/ha + organic fertilizer 3750 kg/ha (TF1), shellfish amendment 750 kg/ha + organic fertilizer 7500 kg/ha (TF2), shellfish amendment 1500 kg/ha + organic fertilizer 3750 kg/ha (TF3), shellfish amendment 1500 kg/ha + organic fertilizer 7500 kg/ha (TF4), shellfish amendment 2250 kg/ha + organic fertilizer 3750 kg/ha (TF5), shellfish amendment 2250 kg/ha + organic fertilizer 7500 kg/ha (TF6).

**Table 5:** Effects of shellfish amendment and organic fertilizer on tea yield (100-bud weight, moisture content of new shoots, budding density, and fresh leaves yield).

Treatment	100-Bud Weight (g)	Moisture Content of New Shoots	Budding Density (m^2^)	Fresh Leaves Yield (kg/ha)
CK	22.10 ± 0.73 b	0.51 ± 0.02 a	208.54 ± 9.24 c	460.42 ± 24.65 c
TF1	22.86 ± 1.52 b	0.51 ± 0.01 a	211.33 ± 4.26 c	483.66 ± 31.14 c
TF2	25.36 ± 1.26 a	0.51 ± 0.01 a	233.33 ± 5.02 ab	561.86 ± 29.56 ab
TF3	23.96 ± 1.45 b	0.53 ± 0.02 a	227.66 ± 2.51 b	545.62 ± 33.07 b
TF4	27.40 ± 0.55 a	0.53 ± 0.04 a	238.66 ± 14.63 ab	554.45 ± 22.35 b
TF5	23.86 ± 0.47 b	0.54 ± 0.02 a	246.66 ± 10.07 a	578.52 ± 18.66 ab
TF6	26.73 ± 0.82 a	0.51 ± 0.01 a	248.33 ± 3.15 a	584.02 ± 26.93 a

Data represent means ± standard deviation. Different lowercase letters in the table indicate significant differences between different amendment treatments (*p* < 0.05). Note: no shellfish amendment or organic fertilizer (CK), shellfish amendment 750 kg/ha + organic fertilizer 3750 kg/ha (TF1), shellfish amendment 750 kg/ha + organic fertilizer 7500 kg/ha (TF2), shellfish amendment 1500 kg/ha + organic fertilizer 3750 kg/ha (TF3), shellfish amendment 1500 kg/ha + organic fertilizer 7500 kg/ha (TF4), shellfish amendment 2250 kg/ha + organic fertilizer 3750 kg/ha (TF5), shellfish amendment 2250 kg/ha + organic fertilizer 7500 kg/ha (TF6).

**Table 6:** Effects of shellfish amendment and organic fertilizer on growth and nutrient concentration of tea (SPAD, N, P and K concentration).

Treatment	SPAD	N Concentration (g/kg)	P concentration (g/kg)	K Concentration (g/kg)
CK	49.62 ± 2.28 b	33.65 ± 3.23 b	2.47 ± 0.10 b	11.08 ± 1.59 b
TF1	56.56 ± 2.23 a	33.94 ± 3.15 b	2.72 ± 0.14 a	11.73 ± 1.03 a
TF2	57.82 ± 3.04 a	35.08 ± 2.73 ab	2.55 ± 0.27 a	11.32 ± 0.75 ab
TF3	55.94 ± 2.70 a	35.94 ± 0.27 ab	2.64 ± 0.33 a	11.36 ± 0.80 ab
TF4	56.10 ± 3.78 a	35.69 ± 1.33 ab	2.55 ± 0.11 a	11.55 ± 0.17 ab
TF5	56.63 ± 4.04 a	35.22 ± 2.05 ab	2.63 ± 0.27 a	12.06 ± 0.96 a
TF6	58.29 ± 1.81 a	36.27 ± 4.73 a	2.86 ± 0.08 a	12.15 ± 0.80 a

Data represent means ± standard deviation. Different lowercase letters in the table indicate significant differences between different amendments treatments (*p* < 0.05). Note: no shellfish amendment or organic fertilizer (CK), shellfish amendment 750 kg/ha + organic fertilizer 3750 kg/ha (TF1), shellfish amendment 750 kg/ha + organic fertilizer 7500 kg/ha (TF2), shellfish amendment 1500 kg/ha + organic fertilizer 3750 kg/ha (TF3), shellfish amendment 1500 kg/ha + organic fertilizer 7500 kg/ha (TF4), shellfish amendment 2250 kg/ha + organic fertilizer 3750 kg/ha (TF5), shellfish amendment 2250 kg/ha + organic fertilizer 7500 kg/ha (TF6).

**Table 7:** Effects of shellfish amendment and organic fertilizer on tea quality (tea polyphenols, free amino acids, tea polyphenols to free amino acids ratio, caffeine, and water extracts).

Treatment	Tea Polyphenols (mg/g)	Free Amino Acids (mg/g)	Tea Polyphenols to Free Amino Acids Ratio	Caffeine (mg/g)	Water Extracts (mg/g)
CK	165.34 ± 4.87 b	32.18 ± 3.21 b	5.13 ± 0.16 a	30.41 ± 1.26 b	398.76 ± 10.72 b
TF1	168.51 ± 5.61 b	32.64 ± 2.41 b	5.16 ± 0.22 a	31.56 ± 0.63 b	407.24 ± 24.36 b
TF2	166.32 ± 5.72 b	35.55 ± 3.24 ab	4.67 ± 0.12 b	31.24 ± 0.84 b	408.65 ± 20.17 b
TF3	175.83 ± 4.12 ab	34.71 ± 2.38 b	5.06 ± 0.33 a	31.49 ± 1.12 b	420.23 ± 15.64 ab
TF4	180.76 ± 3.61 ab	38.26 ± 3.61 ab	4.72 ± 0.21 b	32.57 ± 2.15 b	415.74 ± 20.31 ab
TF5	193.82 ± 2.89 a	40.59 ± 1.85 a	4.77 ± 0.14 b	32.82 ± 3.24 b	430.61 ± 23.15 a
TF6	197.74 ± 5.34 a	41.67 ± 2.34 a	4.74 ± 0.23 b	36.53 ± 2.74 a	436.28 ± 36.24 a

Data represent means ± standard deviation. Different lowercase letters in the table indicate significant differences between different amendments treatments (*p* < 0.05). Note: no shellfish amendment or organic fertilizer (CK), shellfish amendment 750 kg/ha + organic fertilizer 3750 kg/ha (TF1), shellfish amendment 750 kg/ha + organic fertilizer 7500 kg/ha (TF2), shellfish amendment 1500 kg/ha + organic fertilizer 3750 kg/ha (TF3), shellfish amendment 1500 kg/ha + organic fertilizer 7500 kg/ha (TF4), shellfish amendment 2250 kg/ha + organic fertilizer 3750 kg/ha (TF5), shellfish amendment 2250 kg/ha + organic fertilizer 7500 kg/ha (TF6).

## Data Availability

The data presented in this study are available on request from the corresponding author.
